# Rethinking Parkinson’s disease genetics in the precision medicine era: why genomic diversity matters?

**DOI:** 10.3389/fgene.2025.1750113

**Published:** 2026-01-12

**Authors:** Camilla Teixeira Pinheiro Gusmão, Giselli Scaini, Everton Ferreira de Souza, Rafael Antônio Vicente Lacerda, Matheus de Almeida Costa, Raja Mehanna, João Quevedo, Howard Lopes Ribeiro Junior

**Affiliations:** 1 Center for Interventional Psychiatry, Faillace Department of Psychiatry and Behavioral Sciences at McGovern Medical School, The University of Texas Health Science Center at Houston (UTHealth), Houston, TX, United States; 2 Translational Psychiatry Program, Faillace Department of Psychiatry and Behavioral Sciences at McGovern Medical School, The University of Texas Health Science Center at Houston (UTHealth), Houston, TX, United States; 3 Center of Excellence on Mood Disorders, Faillace Department of Psychiatry and Behavioral Sciences at McGovern Medical School, The University of Texas Health Science Center at Houston (UTHealth), Houston, TX, United States; 4 Neuroscience Graduate Program, The University of Texas MD Anderson Cancer Center UTHealth Graduate School of Biomedical Sciences, Houston, TX, United States; 5 Clinics Hospital, Faculty of Medicine, University of São Paulo, SãoPaulo, Brazil; 6 Josué Monteiro de Abreu Primary Healthcare Unit, Ministry of Health of Brazil, Maranguape, Ceará, Brazil; 7 Department of Gastroenterology, Faculty of Medicine, University of São Paulo (USP), São Paulo, Brazil; 8 UTMOve, Department of Neurology, The University of Texas Health Science Center at Houston (UTHealth), Houston, TX, United States; 9 Translational Psychiatry Laboratory, Graduate Program in Health Sciences, University of Southern Santa Catarina (UNESC), Criciúma, Santa Catarina, Brazil; 10 Center for Research and Drug Development (NPDM), Federal University of Ceará, Fortaleza, Ceará, Brazil; 11 Post-Graduate Program in Pathology, Federal University of Ceará, Fortaleza, Ceará, Brazil; 12 Post-Graduate Program in Translational Medicine, Federal University of Ceará, Fortaleza, Ceará, Brazil

**Keywords:** admixture populations, genetic, genomic diversity, Parkinson’s disease, precision medicine

## Introduction

Parkinson’s disease (PD) is a progressive neurodegenerative disorder characterized primarily by motor symptoms, including bradykinesia, rigidity, and resting tremor, and is frequently accompanied by a spectrum of non-motor manifestations such as cognitive decline, mood disorders, and autonomic dysfunction (Tanner and Ostrem, 2024; Chaudhary et al., 2025). Globally, PD affects more than 10 million individuals, and its prevalence is projected to double by 2040. The incidence of PD increases significantly with age, with men generally facing a slightly higher risk than women. However, regional differences suggest that genetic, environmental, and lifestyle factors together influence disease susceptibility (Su et al., 2025).

Despite decades of research, the etiology of PD remains only partially understood (Dong-Chen et al., 2023). While environmental exposures, such as pesticides and head trauma, contribute to risk, genetic factors are recognized as core determinants of disease onset and progression. Mutations in genes like *SNCA*, *LRRK2*, and *GBA*, among others, have highlighted molecular pathways involved in α-synuclein aggregation, lysosomal dysfunction, and mitochondrial impairment (Pang et al., 2019; Periñán et al., 2022). However, these variants account for only a fraction of PD cases, and most genetic susceptibility is regulated by common, low-effect polymorphisms whose influence is modulated by complex gene-gene and gene-environment interactions (Pitz et al., 2024).

Today, the genomic landscape of PD is still highly heterogeneous across populations. Most large-scale studies, including genome-wide association studies (GWAS), have focused predominantly on individuals of European ancestry. While Asian populations are represented to some extent, particularly through studies from India and China, Latin America, Africa, Central Asia and Southeast Asia remain underrepresented (Schumacher-Schuh et al., 2022). This bias limits the generalizability of identified risk loci and polygenic risk scores (PRSs), as allelic frequencies, linkage patterns, and regulatory architectures differ substantially across ancestries (Saffie Awad et al., 2025). The exclusion of diverse genomes, therefore, creates obstacles not only in the discovery of ancestry-specific risk and protective variants but also constrains the development of accurate diagnostic tools, predictive models, and tailored therapies.

**FIGURE 1:**
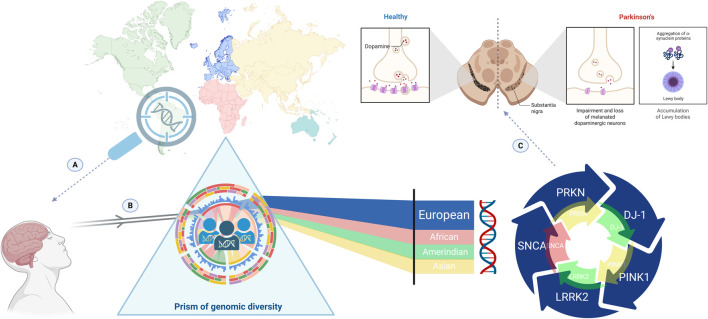
The prism of genomic diversity and Parkinson’s disease (PD) genetics in the precision medicine era. The magnifying glass highlights genomic investigation in admixed and underrepresented populations, emphasizing regions historically neglected in PD genetic research **(A)**. When genetic data from diverse ancestries pass through the prism of genomic diversity **(B)**, differences in allele frequencies, linkage disequilibrium patterns, and ancestry-specific genomic architectures are revealed, generating a broader and more representative spectrum of insight compared with the predominantly European-centered approach. The downstream impact of this imbalance is illustrated at the neurobiological level **(C)**, where incomplete genetic representation constrains risk prediction, variant interpretation, and the understanding of PD pathophysiology, ultimately limiting the application of precision medicine across global populations.

Including underrepresented and admixed populations in PD genetics research is essential for producing more accurate and equitable science (Figure 1). Regions with complex admixture patterns, such as Brazil, Colombia, Mexico, and South Africa, represent unique natural laboratories for elucidating novel disease mechanisms and gene-environment interactions (Marconi et al., 2025). By capturing the full spectrum of human genomic diversity, researchers can refine pathogenicity assessments, enhance the accuracy of genetic counseling, and identify population-specific therapeutic targets.

In this Opinion, we emphasize that expanding genetic research on PD to include admixed and underrepresented populations, such as Latin Americans, Africans, Middle Eastern, is both an ethical imperative and a scientific necessity. Inclusion of these populations can reshape our understanding of PD pathogenesis, refine global risk prediction, and accelerate the growth of equitable, ancestry-aware therapeutic strategies. The exclusion of diverse genetic backgrounds not only distorts risk interpretation but also constrains the future of personalized neurology for a large portion of the world’s population.

## The genetic landscape of Parkinson’s disease: current advances and persistent gaps

The genetic architecture of PD encompasses both rare, high-penetration variants in genes such as *SNCA*, *LRRK2*, *PINK1*, *PRKN*, and *DJ-1*, and common, low-effect polymorphisms identified through GWAS approaches (Bandres-Ciga et al., 2020; Blauwendraat et al., 2020). While pathogenic mutations in LRRK2 and GBA account for a fraction of familial and sporadic PD cases, many risk-associated variants lie within noncoding regions, modulating gene expression through subtle regulatory mechanisms (Ito and Utsunomiya-Tate, 2023).

Recent studies reinforce the importance of SNCA polymorphisms, especially rs356165, rs356219, and rs11931074, across populations, although their effect sizes differ by ancestry (Mohammadi et al., 2025). Such variability likely reflects differences in linkage disequilibrium structure, chromatin organization, and DNA methylation landscapes, suggesting that epigenetic regulation of SNCA may be ancestry-dependent. Despite these findings, most data originate from European cohorts, whose data dominate GWAS repositories and meta-analyses. Consequently, the transferability of these results to admixed genomes, such as those found in Latin American, African, or South Asian populations, remains highly uncertain (Saffie Awad et al., 2024b; Saffie Awad et al., 2025).

Santos-Lobato et al. (2021) highlighted this paradox in their review. For example, in Brazil, one of the world’s most genetically diverse nations (Nunes et al., 2025), only 3.2% of PD patients screened had identifiable monogenic mutations, and population admixture was rarely analyzed (Santos-Lobato et al., 2021). Countries with the richest ancestral diversity thus contribute the least to genetic discovery, while results from homogeneous European samples are often generalized to the entire human population. This imbalance undermines the promise of precision medicine in PD. PRSs derived from European datasets commonly lack reproducibility in non-Europeans and in admixed populations. For instance, LRRK2 p.G2019S, prevalent among Ashkenazi Jews and North African Arabs, exhibits low frequency in East Asians but displays variable penetrance in Latin American cohorts. Similarly, GBA variants, critical risk modifiers, show heterogeneous effects across ancestries, influenced by background haplotypes and local selective pressures (Claudino dos Santos et al., 2024).

Populations such as those in Brazil, Colombia, Mexico, and South Africa represent unique genetic mosaics that can shed light on novel biological pathways and protective mechanisms. The persistent underrepresentation of these groups perpetuates misclassification of variants and diagnostic uncertainty. Understanding allele frequencies, linkage patterns, and gene-environment interactions within these populations could refine pathogenicity assessments and enable more accurate genetic counseling. Moreover, epigenetic plasticity within SNCA intronic regions, varying by ancestry, may influence transcriptional dynamics and disease onset, further demonstrating that diversity is not optional but essential for comprehensive genomic interpretation (Sharma et al., 2019).

A compelling illustration of the consequences of underrepresentation comes from another disease, transthyretin amyloidosis (ATTR) (Bharaj et al., 2025). As discussed by Bharaj et al. (2025), the TTR V122I variant, carried by approximately 3.4% of African Americans, was long neglected in cardiogenetic research due to Eurocentric sampling. Only when African ancestry populations were investigated did the variant’s pathogenic role in heart failure become evident, ultimately leading to the development of targeted therapies such as Tafamidis. This case demonstrates how inclusion can drive therapeutic innovation when diversity is integrated into discovery pipelines: new biological pathways emerge, and medical inequities begin to narrow. We see that a similar transformation is needed in the context of PD (Bharaj et al., 2025).

Expanding genetic studies to underrepresented and admixed populations could reveal ancestry-specific modulators of α-synuclein aggregation, lysosomal function, or mitochondrial dynamics, all of which are central to neurodegeneration. Countries with mixed ancestries serve as natural laboratories for exploring gene-environment interactions. For example, the Brazilian population’s heterogeneous admixture, predominantly European in the South, mixed African and Indigenous in the North and Northeast, mirrors global genetic variation and offers a fertile ground for trans-ancestral mapping. Yet, as reported by Santos-Lobato et al. (2021), fewer than 100 Brazilian PD patients have been genetically characterized to date.

Incorporating such populations would refine PRS calibration and variant interpretation. A variant labeled as “pathogenic” in a European cohort may be benign, or even protective, in an admixed genome. Without ancestry-adjusted databases, clinical misinterpretations are inevitable, perpetuating inequities in diagnosis and access to treatment (Wang et al., 2024). Thus, expanding PD genomics to include underrepresented populations is not merely an act of inclusion, it is a scientific necessity for the validity and global applicability of genetic medicine.

## Ethical and scientific imperatives for global inclusion

The lack of diversity in PD genetic research remains a significant barrier to advancing the field (Schumacher-Schuh et al., 2022). More than 75% of participants in PD GWAS are of European ancestry, while fewer than 4% represent African, Latin American, or Asian, other than Chinese, ancestry. This imbalance has deep ethical and scientific implications, influencing not only the interpretation of genetic risk but also how diagnostic and therapeutic tools are developed and implemented globally (Mohamed, 2023; Claudino dos Santos et al., 2024).

From an ethical standpoint, exclusion from genomic research translates directly into inequity in knowledge and care of PD. Populations in Latin America, Africa, and Asia bear a growing burden of PD, yet the biological understanding of the disease and the technologies derived from it are built predominantly on genetic architectures that do not represent them (Siddiqi et al., 2025). This Eurocentric bias perpetuates scientific dependency, in which discoveries made in the Global North are generalized to all populations, often leading to misclassification of variants, reduced diagnostic accuracy, and missed opportunities for targeted interventions. True inclusion in PD genetics is not only a matter of representation; it is a prerequisite for fairness in precision medicine (Lange et al., 2025).

Scientifically, the lack of representation has measurable effects. PRS for PD, derived almost exclusively from European datasets, shows reduced predictive accuracy when applied to admixed genomes such as those found in Brazil, Colombia, or South Africa (Fatumo et al., 2023). The same holds true for pathogenic variant mutations in LRRK2, GBA, or SNCA, which show distinct frequencies and penetrance across ancestries, yet the lack of population-specific data limits our ability to interpret these differences accurately. Without diverse genomic reference panels, risk prediction tools remain incomplete, and potential ancestry-specific disease mechanisms remain undiscovered (Abreu et al., 2016).

Recent initiatives, such as the Global Parkinson’s Genetics Program (GP2) (Global Parkinson’s Genetics Program, 2021; Kaufmann et al., 2025), represent an important step toward global inclusivity by integrating cohorts from Africa, Asia, and Latin America. However, disparities in funding, infrastructure, and training still limit full participation. Many institutions in the Global South lack access to next-generation sequencing platforms, bioinformatics pipelines, or stable research financing. Consequently, even when samples are collected locally, data analysis and publication often occur abroad, a phenomenon that risks perpetuating “genomic extractivism”, where genetic resources are exported without reciprocal benefit for the populations involved (Helmy et al., 2016).

To address this imbalance, it is necessary to strengthen local capacity in these underrepresented regions. Establishing regional biobanks, training programs in genetic epidemiology and bioinformatics, and supporting South-South scientific networks would enable scientists in underrepresented regions to lead genomic studies. Building robust genomic infrastructure in countries like Brazil, Mexico, and South Africa would not only expand the ancestral spectrum of PD research but also stimulate the discovery of novel variants and gene-environment interactions relevant to local populations. Moreover, integrating environmental, socioeconomic, and epigenetic dimensions into PD genetic research is essential to understand disease heterogeneity across regions better. Populations in tropical and developing regions are exposed to distinct environmental factors, including pesticide exposure, infectious diseases, nutritional patterns, and occupational stressors that may interact with genetic susceptibility to influence disease onset and progression (Polito et al., 2016). Thus, studies combining genomic data with environmental and lifestyle variables could unveil unique mechanisms of neurodegeneration, improving risk stratification and therapeutic design across ancestries.

Community engagement and transparent governance are also essential (Saffie-Awad et al., 2024a). Genomic research should not be extractive but participatory, ensuring that local communities understand and benefit from discoveries arising from their data. Informed consent processes should reflect local cultural context, and benefit-sharing mechanisms should help ensure that clinical applications, such as genetic testing panels or risk models, are accessible to those who made their development possible.

Ultimately, expanding PD genetic research to include admixed and underrepresented populations is both a scientific imperative and a moral responsibility (Tan et al., 2024; Tenchov et al., 2025). Diversity enhances discovery by revealing ancestry-specific biological pathways, but it also ensures that the resulting knowledge serves all of humanity. As the field advances toward precision neurology, inclusivity must evolve from a corrective measure into a foundational principle of study design. Only by embracing global genetic diversity can we build risk models, therapeutic strategies, and ethical frameworks that truly reflect the universality of Parkinson’s disease.

## Conclusion

Bridging the diversity gap in PD research requires a translational approach. Large-scale sequencing of underrepresented groups should be integrated with clinical phenotyping and bioinformatic integration. Multi-omics strategies combining genomics, transcriptomics, and metabolomics may help identify ancestry-specific endophenotypes, improving diagnosis and therapeutic targets. Moreover, equitable genomic medicine demands inclusive data governance. Community engagement, ethical consent frameworks, and data-sharing agreements must ensure that benefits derived from genomic research return to the populations that enable them. Only through such reciprocity can the global scientific community achieve both rigor and justice.

Finally, the genetic understanding of PD is at a turning point. While European cohorts have laid the foundation for discovery, the next phase will require incorporating the full spectrum of human diversity. Inclusion of admixed and underrepresented populations is essential for improving accuracy, expanding variant discovery, and strengthening precision-medicine strategies across populations. By expanding research efforts in Latin America, Africa, and Asia, we can uncover population-specific variants, refine precision medicine, and move toward a truly global model of neurogenetic research. In doing so, we fulfill both the scientific and moral mandate of modern genetics: to understand disease in all of humanity, not just a fraction of it.
